# Supplementary material for: The microbiome biomarkers of pregnant women’s vaginal area predict preterm prelabor rupture in Western China
Source: Front Cell Infect Microbiol. 2024 Oct 31;14:1471027. doi: 10.3389/fcimb.2024.1471027 (PMC11560878; doi:10.3389/fcimb.2024.1471027)
Supplement: Supplementary file 1 [file DataSheet1.zip › compare_1/Community/Pieplot/family/group/P.family.pieplot.pdf]

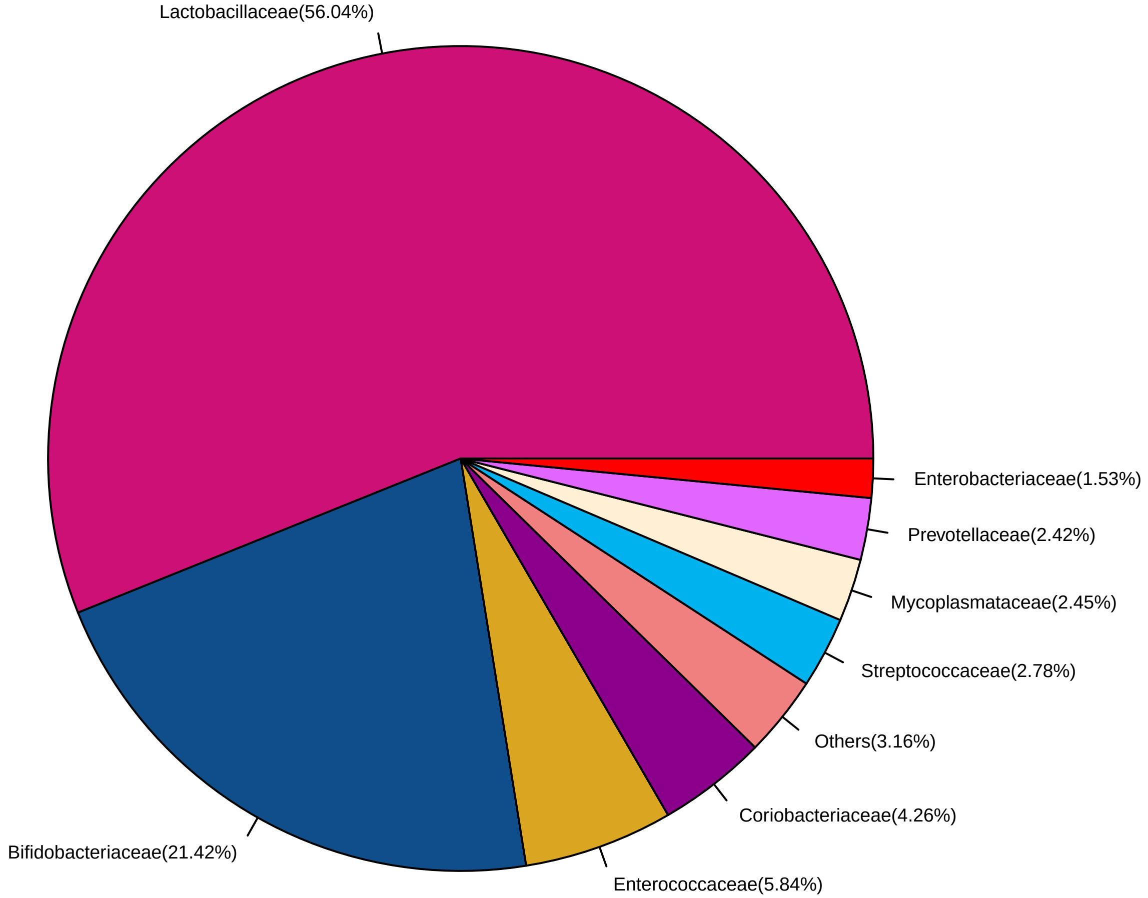
Lactobacillaceae(56.04%)
Enterobacteriaceae(1.53%)
Prevotellaceae(2.42%)
Mycoplasmataceae(2.45%)
Streptococcaceae(2.78%)
Others(3.16%)
Coriobacteriaceae(4.26%)
Enterococcaceae(5.84%)
Bifidobacteriaceae(21.42%)
